# 2200. Impact of COVID-19 Pandemic on Influenza-like Illness (ILI) Experience among Healthcare Workers in Military Treatment Facilities

**DOI:** 10.1093/ofid/ofac492.1819

**Published:** 2022-12-15

**Authors:** Ryan Liberg, Christina Schofield, Stephanie A Richard, Limone Collins, Christina Spooner, Srihari Seshadri, Anuradha Ganesan, Wesley R Campbell, David Hrncir, Tahaniyat Lalani, Tyler Warkentien, Katrin Mende, Ana E Markelz, Catherine M Berjohn, Bruce McClenathan, Jitendrakumar Modi, Alan Williams, Timothy Burgess, Rhonda E Colombo

**Affiliations:** Madigan Army Medical Center, Joint Base Lewis-McChord, Washington; Madigan Army Medical Center Division of Infectious Diseases, Infectious Disease Clinical Research Program, Tacoma, Washington; Infectious Disease Clinical Research Program, Department of Preventive Medicine and Biostatistics, Uniformed Services University of the Health Sciences, Bethesda, MD, USA, Bethesda, MD; Immunization Healthcare Division, Defense Health Agency, Bethesda, Maryland; Immunization Healthcare Division, Defense Health Agency, Bethesda, Maryland; Immunization Healthcare Division, Defense Health Agency, Bethesda, Maryland; Infectious Disease Clinical Research Program, Department of Preventive Medicine and Biostatistics, Uniformed Services University of the Health Sciences, Bethesda, MD, USA, Walter Reed National Military Medical Center, Bethesda, Maryland; Walter Reed National Military Medical Center, Bethesda, Maryland; Carl R. Darnall Army Medical Center/Wilford Hall Ambulatory Surgical Center, Fort Hood, Texas; Naval Medical Center Portsmouth, Portsmouth, Virginia; Naval Medical Center Portsmouth, Portsmouth, Virginia; Infectious Disease Clinical Research Program, Department of Preventive Medicine and Biostatistics, Uniformed Services University of the Health Sciences, Bethesda, MD, USA, Bethesda, MD; Brooke Army Medical Center, San Antonio, Texas; Naval Medical Center San Diego Division of Infectious Diseases, Infectious Disease Clinical Research Program, San Diego, CA; Womack Army Medical Center, Fort Bragg, North Carolina; Naval Health Clinic, Annapolis, MD, Anapolis, Maryland; Uniformed Services University of the Health Sciences, Bethesda, Maryland; Infectious Disease Clinical Research Program, Department of Preventive Medicine and Biostatistics, Uniformed Services University of the Health Sciences, Bethesda, MD, USA, Bethesda, MD; Infectious Disease Clinical Research Program, Department of Preventive Medicine and Biostatistics, Uniformed Services University of the Health Sciences, Bethesda, MD, USA, The Henry M. Jackson Foundation for the Advancement of Military Medicine, Madigan Army Medical Center Division of Infectious Diseases, Tacoma, Washington

## Abstract

**Background:**

Healthcare workers (HCWs) are at heightened risk of exposure to respiratory pathogens, and occupy an important epidemiologic position in the COVID-19 pandemic. PAIVED, a multicenter, multiservice study assessing influenza vaccine effectiveness in the Department of Defense over four consecutive influenza seasons (2018-22), provides an opportunity to describe influenza like illness (ILI) experience and assess the impact of SARS-CoV-2 in HCWs compared to non-HCWs.

**Methods:**

PAIVED participants were randomized to receive either egg-based, cell-based, or recombinant-derived influenza vaccine and then surveyed weekly for ILI. At enrollment, participants provided key demographic data including whether they were HCWs with direct patient contact. ILI was defined *a priori* as 1) having cough or sore throat plus 2) feeling feverish/having chills or having body aches/fatigue. Participants with ILI completed a symptom diary for seven days and submitted a nasal swab for pathogen detection. Study recruitment was conducted from September-January over four consecutive years.

**Results:**

Of 13188 eligible participants enrolled, 4819 (36%) were HCWs. Overall, HCWs were more likely to be female (43% vs 31%), active duty military (86% vs 69%), and to identify as white (61% vs 56%). HCWs more commonly reported ILI than non-HCWs (25% vs 21%, p< 0.01). Of those experiencing ILI, SARS-CoV-2 was identified in a higher proportion of HCWs than non-HCWs (17% vs 12%, p< 0.01). Influenza was isolated in similar proportion of HCWs and non-HCWs (5% vs 4%). Each group reported similar ILI duration and severity (p< 0.01).

**Conclusion:**

In a prior analysis of the 2019-20 PAIVED season, HCWs were more likely than non-HCWs to report ILI, have shorter illness duration, and isolate influenza A (H1N1). The propensity for HCWs to report ILI persisted over the four years. While SARS-CoV-2 emerged as a major pathogen in both groups, HCWs were more likely to have it identified as a cause of ILI, suggesting increased risk of symptomatic SARS-CoV-2 in our HCW population. Influenza incidence was lower than that of SARS-COV-2, and did not differ between HCWs and non-HCWs. Mean duration of illness did not differ between groups over four years; this equalization may relate to the higher incidence of SARS-CoV-2 in HCWs.

**Disclosures:**

**Jitendrakumar Modi, MD**, GlaxoSmithKline: I am a paid speaker for GSK. I do not speak for their flu brand. **Timothy Burgess, MD, MPH**, AstraZeneca: The HJF, in support of the USU IDCRP, was funded to conduct or augment unrelated Phase III Mab and vaccine trials as part of US Govt. COVID19 response.

